# Factors underlying differences in knowledge, explicit stigma and implicit biases towards autism across Hong Kong, the United Kingdom and the United States

**DOI:** 10.1177/13623613241290565

**Published:** 2024-11-02

**Authors:** Yulin Cheng, Patrick Dwyer, Connor Tom Keating

**Affiliations:** 1 The University of Hong Kong, China; 2 University of California Davis, USA; 3 La Trobe University, Australia; 4 University of Birmingham, UK; 5 University of Oxford, UK

**Keywords:** autism spectrum disorders, collectivism, cross-cultural, culture, environmental factors, explicit attitudes, implicit biases, individualism, knowledge, stigma

## Abstract

A growing literature suggests that there is cross-cultural variation in levels of autism-related stigma, which may partially be explained by differences in cultural orientation or autism-related knowledge between countries. This literature has relied heavily on self-report measures of explicit attitudes towards autism; little research has examined implicit biases, and whether these implicit biases vary across cultures. Thus, here we had two aims: (1) to assess the contribution of knowledge and cultural orientation to *both* explicit stigma and implicit biases, and (2) to compare autism-related knowledge, explicit stigma, and implicit biases across university students in Hong Kong (*n* = 119), the United Kingdom (*n* = 120), and the United States (*n* = 122). Replicating prior work, we found that explicit stigma was predicted by less accurate knowledge, lower horizontal collectivism, greater vertical individualism, and higher implicit biases. Implicit biases were *directly* predicted by age and explicit stigma, and *indirectly* predicted by vertical individualism (positively) and horizontal collectivism (negatively) via explicit stigma. Knowledge and explicit stigma differed across countries, even after accounting for covariates: students in Hong Kong displayed less accurate knowledge, and higher explicit stigma towards autism, than those in the United Kingdom and United States. However, implicit biases did not differ between countries.

**Lay abstract:**

Attitudes towards autism vary across countries. Some of this variation could reflect differences in cultural values across countries, or differences in how much people know about autism. Until now, most research on this topic has asked people directly about their attitudes towards interacting with autistic people. As a result, we understand little about why some people unconsciously hold negative attitudes towards autism, and whether these unconscious attitudes vary across countries. We studied explicit attitudes (willingness to interact), implicit attitudes (unconscious beliefs), knowledge about autism, and cultural values in university students from Hong Kong, the United Kingdom and the United States. We found that people were less willing to interact with autistic people if they knew less about autism, aligned with a competitive and hierarchical society (‘vertical individualism’), did not see themselves as part of a collective whose members are equal (less ‘horizontal collectivism’), and if they unconcsciously associated autism with negative attributes. Students in Hong Kong were less willing to interact with autistic people and had less understanding of autism compared to those in the United Kingdom and the United States. Unconscious biases did not differ across countries. Our findings highlight the need to combat misconceptions about autism to improve attitudes towards autistic people, especially in Hong Kong. Unfortunately, our results suggest that acquiring more accurate knowledge may not be sufficient to alter unconscious biases. Further research is needed to determine the factors underlying unconscious biases.

## Introduction

Autistic people^1^ face stigma in their everyday lives (see Turnock et al., 2022). Neurotypicals often form negative first impressions of autistic individuals (Sasson et al., 2017), are less likely to want to spend time with an autistic person as friends (Nevill & White, 2011), and are less likely to want an intimate relationship with an autistic person (Jensen et al., 2016). Indeed, autistic individuals can be dehumanized by their neurotypical peers (Cage et al., 2018). Unfortunately, these attitudes can have a negative impact, resulting in exclusion, bullying, self-stigma (see Han et al., 2022), and poorer mental and physical health for autistic people (e.g. Botha & Frost, 2020; Cage et al., 2018; Keating et al., 2024; Turnock et al., 2022) and their families (see Papadopoulos et al., 2019). Increased awareness of these negative consequences has resulted in a growing number of studies aiming to identify the factors underlying autism-related stigma.

### Factors underlying autism-related stigma

Historically, the majority of global autism research has taken place in Western countries, such as the United States and United Kingdom (Rong et al., 2022; Sweileh et al., 2016). While cultural variation exists within and beyond national borders (Henderson et al., 2013), confining research to a single region/population reduces cultural variability and can lead researchers to take cultural norms for granted, whereas between-group comparisons can illuminate relevant cultural differences (Oyserman, 2017). Thus, cross-cultural comparisons could be useful for identifying the factors underpinning stigma (Keating et al., 2021, 2024).

An emerging body of research suggests that autism-related stigma – measured as self-reported, explicit behavioural intentions towards autistic people – is higher in South Korea (Kim et al., 2022), Japan (Keating et al., 2024; Someki et al., 2018), Lebanon (Gillespie-Lynch et al., 2019; Obeid et al., 2015), and Malaysia (de Vries et al., 2020) than in the United States or United Kingdom. This suggests an opportunity to identify relevant factors that could, in principle, be driving these differences in stigma, such as varying levels of knowledge about autism, or differences in cultural value orientation between these countries (see Gillespie-Lynch et al., 2019; Keating et al., 2024).

One factor that has been found to contribute to autism-related stigma is a lack of, or less accurate knowledge about autism (Gillespie-Lynch et al., 2019; Kim et al., 2022; Kitchin & Karlin, 2021; Kuzminski et al., 2019; Ling et al., 2010; Obeid et al., 2015). Although public awareness of autism may have improved in parts of the world such as Australia (S. C. Jones et al., 2021), Denmark (Jensen et al., 2016), France (Durand-Zaleski et al., 2012), South Korea (Park et al., 2018), the United Kingdom (Cage et al., 2019; Keating et al., 2024), and Canada (G. E. Mitchell & Locke, 2015), even in these countries there is evidence of superficial and outdated knowledge, and persisting misconceptions about autism and autistic people (Jensen et al., 2016; S. C. Jones et al., 2021; Park et al., 2018; White et al., 2019). As argued by Turnock et al. (2022), this ‘limited knowledge . . . has a critical role in the presence of stigmatized views, as an individual is more likely to rely on reductionist labels and stereotypes, dichotomize into them versus us, and ultimately behave in a discriminatory way’ (p. 77). As well as this limited knowledge contributing to higher autism-related stigma, it is important to note that knowledge rooted in certain ideologies may precipitate stigma. For example, knowledge based on the medical model – which frames autism as a pathology – is linked to higher levels of autism-related stigma (Gillespie-Lynch et al., 2017), while support for the neurodiversity movement is associated with reduced stigma (Kim & Gillespie-Lynch, 2023). Finally, research has also found that increasing autism knowledge through training can lead to some reductions in stigma among university students (Gillespie-Lynch et al., 2015; Jones, DeBrabander, & Sasson, 2021a; Jones, Morrison, et al., 2021; Obeid et al., 2015; Saade et al., 2021), especially when training programmes are developed by, or in collaboration with autistic people (Gillespie-Lynch et al., 2022). Together, this work provides convincing evidence that autism-related stigma may, at least in part, be underpinned by incomplete or inaccurate knowledge.

Another factor that could underpin differences in levels of autism-related stigma is cultural value orientation. One conceptualization of cultural value orientation decomposes it into two independent, orthorgonal dimensions: individualism-collectivism and verticality-horizontality. While individualistic cultures place emphasis on independence and distinctiveness, collectivistic cultures emphasize community interdependence and shared group norms (Triandis, 1988). Relatedly, while horizontal cultures value equality, vertical cultures value hierarchy, among its members (Shavitt et al., 2006; Singelis et al., 1995; Triandis & Gelfand, 1998). A growing body of evidence suggests that autism-related stigma is higher in collectivistic than in individualistic cultures (e.g. in Japan, Lebanon, South Korea than in the United Kingdom or the United States; Gillespie-Lynch et al., 2019; Kim et al., 2022; Obeid et al., 2015; Someki et al., 2018), and that higher verticality is linked to greater stigma towards autistic and neurodivergent people (Gillespie-Lynch et al., 2019; Rao et al., 2010).

Notably, however, studies that have broken down the individualism-collectivism and verticality-horizontality dimensions into their constituent elements (vertical individualism, horizontal individualism, vertical collectivism, horizontal collectivism; Triandis & Gelfand, 1998) have found that heightened vertical individualism (seeing oneself as autonomous within a justly hierarchical system) is linked to elevated self-reported stigma towards autistic and neurodivergent people (Gillespie-Lynch et al., 2019; Kim et al., 2022; Rao et al., 2010). Horizontal collectivism (seeing oneself as part of a collective whose members are equal), on the other hand, was linked to *reduced* stigma (Gillespie-Lynch et al., 2019; Kim et al., 2022).

Thus, a growing body of work suggests that there is cross-cultural variation in levels of autism-related stigma, which may be explained (at least in part) by differences in knowledge or differences in cultural orientation between countries. A limitation of this previous work, however, is that it has relied heavily on self-report measures of stigma, investigating explicit attitudes towards autism (e.g. through Social Distance Scales (SDS); Gillespie-Lynch et al., 2019; Kim et al., 2022; Obeid et al., 2015; Someki et al., 2018). Such explicit biases are heavily influenced and controlled by social desirability (Dovidio et al., 1997; Henderson et al., 2012; Stier & Hinshaw, 2007), potentially obscuring underlying or unconscious biases, which persist even among raters who report no explicit biases (see Dickter et al., 2020; Hinshaw & Stier, 2008). To the best of our knowledge, prior research has compared levels of *both* explicit and implicit attitudes towards autism across only two countries (namely, South Korea and the United States; Kim et al., 2023). This study found that both forms of stigma were higher in South Korea than in the United States and elucidated several factors that may predict implicit biases. For example, Kim and colleagues (2023) identified that, while older age and reduced social dominance orientation predicted higher implicit biases in South Korea, the absence of a (nuclear) autistic family member predicted higher implicit biases in the United States. At present, further research is necessary to investigate cross-cultural differences in, and the factors underpinning explicit *and* implicit biases towards autism across additional countries and cultures. Such investigations may shed light on how to minimize both explicit and more deeply ingrained implicit biases, and facilitate the identification of priority regions and needs for anti-stigma interventions.

### The current study

In this study, we first aimed to determine the contribution of knowledge and cultural orientation (among other factors) to *both* explicit stigma and implicit biases towards autism, and second, to compare levels of knowledge, explicit stigma, and implicit biases across Hong Kong, the United Kingdom, and the United States, after controlling for relevant covariates. We selected these countries for three key reasons: (1) their varied cultural orientations enable us to examine whether different values contribute to explicit stigma, implicit biases, and knowledge (by providing sufficient statistical variability); (2) autism-related stigma and knowledge remain understudied in Hong Kong; and (3) although the United Kingdom and the United States are often used as comparison groups in research (e.g. de Vries et al., 2020; Gillespie-Lynch et al., 2019; Kim et al., 2022; Obeid et al., 2015; Someki et al., 2018), no studies have directly compared these factors between them. To fulfil our aims, university students from Hong Kong, the United Kingdom and the United States completed a number of questionnaires and an autism-related Implicit Association Test (IAT; Jones, DeBrabander, & Sasson, 2021a). In line with previous findings (e.g. Gillespie-Lynch et al., 2019; Keating et al., 2024; Kim et al., 2022; Obeid et al., 2015; Someki et al., 2018), we first predicted that those with greater knowledge about autism, higher horizontal collectivism, and lower vertical individualism would exhibit lower levels of explicit autism-related stigma. Second, we hypothesized that levels of explicit stigma and implicit biases would be higher in Hong Kong than in the United Kingdom and United States for several reasons: (1) previous studies have shown widespread autism-related stigma in Hong Kong, with about 75% of caregivers reporting such experiences (Ng & Ng, 2022); (2) acceptance of inequality is higher in Hong Kong (verticality score = 68, compared to 35 in the United Kingdom and 40 in the United States; Hofstede, 1984, 2001), which is linked to increased stigma towards autistic and neurodivergent people (e.g. Gillespie-Lynch et al., 2019; Rao et al., 2010); and (3) Hong Kong’s strong focus on social conformity, neoliberal values, and deficit-based accounts of autism (Cheng et al., 2023; Grinker, 2020; Thomas, 2021), and limited exposure to the neurodiversity movement (Cheng et al., 2023; Kim & Gillespie-Lynch, 2023; Turnock et al., 2022) may further amplify stigma.

## Method

This study was approved by the Science, Technology, Engineering and Mathematics ethics committee at the University of Birmingham (ERN_21-1732), and the Human Research Ethics Committee from the University of Hong Kong (EA210523). The study was conducted in accordance with the principles of the revised Helsinki declaration.

### Participants

In total, 368 university students took part in our study (*N* = 125 Hong Kong, *N* = 120 United Kingdom, *N* = 123 United States). These participants were recruited by advertising the study via University of Hong Kong mailing lists (Hong Kong) and through Prolific (the United Kingdom, the United States) between May 2022 and July 2022. Seven participants were excluded as their IAT data did not meet criteria for inclusion (further details below; Greenwald et al., 2003). As such, our final sample comprised 361 university students; 119 from Hong Kong, 120 from the United Kingdom, and 122 from the United States. All participants were fluent in English, eliminating the need to translate the study materials into Chinese. This allowed us to maintain consistency in the meaning of the questionnaire items and IAT terms, thus ensuring that any differences in stigma, implicit biases, or knowledge arose due to true group differences rather than subtle language discrepancies. Information on participants’ age, gender, course level, course type, and student type across each of these countries can be seen in Table 1. Participants’ ethnicities are reported in Supplementary Materials A.

**Table 1.** Participant demographics. For age, the mean is presented with the standard deviation in parentheses. For gender, course level, course type, and student type, the number of participants within each group is presented.

Characteristic	Hong Kong (*n* = 119)	United Kingdom (*n* = 120)	United States (*n* = 122)
**Age**	22.26 (4.36)	28.74 (10.93)	26.02 (8.21)
**Gender**	75 Cisgender female, 38 Cisgender male, 1 Non-binary/third gender 5 Prefer not to say	79 Cisgender female, 37 Cisgender male, 2 Non-binary/third gender, 1 Other, 1 Prefer not to say	67 Cisgender female, 48 Cisgender male, 3 Non-binary/third gender, 2 Transgender female, 2 Other
**Course Level**	88 Undergraduate, 31 Postgraduate	78 Undergraduate, 41 Postgraduate	89 Undergraduate, 33 Postgraduate
**Course Type**	107 Full-time, 12 Part-time	91 Full-time, 29 Part-time	91 Full-time, 31 Part-time

### Materials and procedure

After providing informed consent, participants completed an online survey using the Qualtrics survey platform (Qualtrics, 2005). The survey began with a series of demographic questions, including participant age, gender, ethnicity, country of birth, country of residence, course level (e.g. undergraduate, postgraduate), course discipline, and student type (e.g. local, international, exchange). Following this, participants completed three previously validated questionnaires: the SDS (Gillespie-Lynch et al., 2019), the Participatory Autism Knowledge Measure (PAK-M; Kim et al., 2022), and the Culture Orientation Scale (Triandis & Gelfand, 1998). Finally, participants completed an autism-related IAT (Jones, DeBrabander, & Sasson, 2021a) on Gorilla.sc (an online experiment platform). All participants completed the study in English.

#### Explicit stigma

Here, we employed a version of the SDS (originally, Bogardus, 1933), adapted by Gillespie-Lynch et al. (2015) for the context of autism. In this 11-item self-report questionnaire, participants rate their willingness to engage with an autistic individual across various levels of intimacy (e.g. ‘I would be willing to spend an evening socializing with an autistic person’) on a 5-point Likert-type scale from ‘Strongly disagree’ to ‘Strongly agree’. Stigma scores on this scale range from −22 to 22 with higher scores indicating higher stigma. Notably, neutral or negative scores should not be interpreted as an absence of stigma (see comments on a similar measure by S. C. Jones (2022)). The SDS boasts strong internal consistency in previous work (α = 0.88–0.89; Gillespie-Lynch et al., 2019), and in this study (α = 0.92, Hong Kong α = 0.87, United Kingdom α = 0.92, United States α = 0.92).

#### Knowledge about autism

The PAK-M is a self-report questionnaire assessing knowledge about autism (Kim et al., 2022). The PAK-M was adapted from the Autism Awareness Survey (Stone, 1987) and was developed with autistic input to reflect changing knowledge and research (Kim et al., 2022). In this questionnaire, participants are required to respond to 29 true-or-false statements (e.g. ‘Autism can be diagnosed as early as 18 months’, ‘Autistic children grow up to be autistic adults’) on a 5-point scale ranging from 1 = ‘Definitely False’ to 5 = ‘Definitely True’. Higher scores represent more accurate and current knowledge about autism. Previous works, and our analyses here, demonstrate that the PAK-M has high internal consistency (Kim et al., 2022: α = 0.71; here: α = 0.80, Hong Kong α = 0.72, United Kingdom α = 0.76, United States α = 0.85).

#### Cultural orientation

The *Culture Orientation Scale* is a 16-item questionnaire that assesses four components of cultural orientation: vertical individualism (e.g. ‘When another person does better than I do, I get tense and aroused’), vertical collectivism (e.g. ‘It is important to me that I respect the decisions made by my groups’), horizontal individualism (e.g. ‘I often do my own thing’), and horizontal collectivism (e.g. ‘If a coworker gets a prize, I would feel proud’; Triandis & Gelfand, 1998). To complete this questionnaire, participants are required to respond on a 9-point scale ranging from 1 = ‘Never or definitely no’ to 9 = ‘Always or definitely yes’. A score for each of the four components is calculated by summing the respones for the relevant items. To facilitate interpretability of one of our plots, we also computed an ‘acceptance of inequality variable’ by subtracting each individual’s total horizontal score from their total vertical score (as in Papadopoulos and colleagues, 2019). The Culture Orientation Scale has been shown to have acceptable internal consistency in previous work (e.g. Gillespie-Lynch et al., 2019: α = 0.75–0.79), and here (α = 0.72, Hong Kong α = 0.74, United Kingdom α = 0.73, United States α = 0.70).

#### Implicit biases

IATs (Greenwald et al., 1998) are used to probe automatic associations between cognitive concepts (e.g. Black, White) and positive or negative attributes (e.g. good, bad). In this study, we developed an IAT based on that from Jones, DeBrabander, and Sasson (2021a) to examine whether participants across our sites unconsciously associate autism diagnostic labels with pleasant or unpleasant personal attributes. The target words (see Table 2) used for each category were taken from Jones, DeBrabander, and Sasson (2021a).

**Table 2.** Implicit Association Test categories and words.

Category	Stimulus words
Diagnosis	
Autism spectrum	Autistic, Autism, Asperger’s
Typically developing	Normal, Neurotypical, No diagnosis
Personality traits	
Pleasant	Nice, friendly, likeable, safe, popular, honest, compassionate, independent
Unpleasant	Awkward, creepy, weird, dangerous, antisocial, needy, unpredictable, helpless

Following the procedures of Jones, DeBrabander, and Sasson (2021a), our IAT task used a five-block design (see Table 3.). In the first block, participants categorized words (e.g. ‘Neurotypical’, ‘Autistic’) based on diagnostic concepts (‘Typically Developing’ and ‘Autism Spectrum’) presented on the left and right sides of the screen. Participants pressed the ‘e’ key to categorize a term as ‘Typically Developing’ or the ‘i’ key to categorize it as ‘Autism Spectrum’. The second block focused on personal attributes, with participants categorizing words (e.g. ‘Friendly’, ‘Awkward’) as either ‘Pleasant’ or ‘Unpleasant’. In the third block, the concept and attribute categories were displayed simultaneously in a prejudice consistent manner (e.g. ‘Typically Developing or Pleasant’ on the left of the screen, ‘Autism Spectrum or Unpleasant’ on the right of the screen), and participants categorized both concept and attribute words one at a time as they appeared. The fourth block was identical to the first block, except the positioning of the two concepts was reversed, with participants pressing the ‘e’ key to categorize a word as ‘Autism Spectrum’ and the ‘i’ key to categorize a word as ‘Typically Developing’. In the fifth block, the diagnostic concept and attribute terms were displayed simultaneously in a prejudice inconsistent manner (e.g. ‘Autism Spectrum or Pleasant’ on the left, ‘Typically Developing or Unpleasant’ on the right), and again participants categorized stimulus words into their respective categories.

**Table 3.** Block order to autism Implicit Association Test.

Block order	Type of judgement	Left side	Right side
1	Diagnostic discrimination	Typically developing	Autism spectrum
2	Personality trait discrimination	Pleasant	Unpleasant
3	Prejudice consistent combination	Typically developing or pleasant	Autism spectrum or unpleasant
4	Diagnostic discrimination, reveresed order	Autism spectrum	Typically developing
5	Prejudice inconsistent combination	Autism spectrum or pleasant	Typically developing or unpleasant

Within blocks 1, 2, and 4 (the learning phases), each word was displayed three times in the centre of the screen in a random order, with each word appearing once before any repeats occurred. For blocks 3 and 5 (testing blocks), each word was displayed once in the centre of the screen in a random order over 22 trials. The inter-trial interval was 100 ms. Scoring for the IAT is based on the D-score, representing the standardized difference in response times for prejudice inconsistent and prejudice consistent pairings (Greenwald et al., 2003); positive scores indicate a relationship between a concept and negative attributes. The IAT showed high internal consistency across all five blocks in this study (overall α ⩾ 0.74; Hong Kong mean α = 0.72, UK mean α = 0.84, US mean α = 0.86), and in previous work (α ⩾ 0.87; Jones, DeBrabander, & Sasson, 2021a).

Raw response latencies were obtained for the IAT and inspected to determine whether any trials or participants exceeded common guidelines for inclusion (e.g. remove trials where response latencies >10,000 ms or <400 ms, remove participants that have latencies <300 ms on more than 10% of trials, etc.; Greenwald et al., 2003). This resulted in the removal of seven participants. Following this, D-scores were calculated using the revised scoring method outlined in Greenwald et al. (2003) and Jones, DeBrabander, and Sasson (2021a): participants’ mean response latencies for the prejudice consistent block were subtracted from those of the prejudice inconsistent block. These difference scores were then divided by the standard deviation of participants’ (own) response latencies across the two blocks, thus providing a standardized score that accounts for individual differences in response latencies. Scores significantly higher than zero indicate a significant association between autism and negative attributes; scores significantly lower than zero indicate a significant association between autism and positive attributes.

### Community involvement

This project comprises a collaboration between autistic and non-autistic researchers. Both autistic and non-autistic researchers were involved in the conceptualization and design of the study, data collection and analysis, and in writing up the manuscript. Moreover, following participatory research guidelines (Fletcher-Watson et al., 2019; Keating, 2021), the researchers also consulted autism community members in their local country (in Hong Kong, the United Kingdom, and the United States, respectively) during the design-phase of the project.

### Transparency and openness

All data files, data analysis scripts, and experimental tasks are openly available at https://osf.io/7cd6g/?view_only=f9e6d0e3375a44fa9f2e5a3767b58523. Our primary analyses were conducted in R Studio (version 2023.06.2) and our exploratory mediation analyses were conducted in JASP (version 0.17.2.1). Notably, we found significant cross-cultural variation in autism-related knowledge and explicit stigma both when we controlled relevant covariates (see Results) and when we did not, thus affording us confidence in our findings.

## Statistical analyses

In the following section, we first determined whether levels of explicit stigma and implicit biases towards autism significantly differed from zero in the aggregated sample, using Wilcoxon signed-rank tests (with the *wilcox.test()* function). Second, we identified the factors contributing to explicit stigma, implicit biases, and knowledge within this aggregated sample using multiple regression (with the *lm()* function) and non-parametric multiple regression (with the *gam()* function). Examining these relationships in the aggregated sample allowed us to capture a wide range of variation in cultural orientation and autism-related knowledge, thereby increasing our ability to detect significant relationships (Keating et al., 2021). By identifying the significant predictors of our dependent variables, we could control for these factors in our cross-country comparisons, ensuring any observed differences were not confounded by variations in these predictors across countries (e.g. different cultural orientations, different levels of knowledge, different ages or genders). Finally, we compared levels of explicit stigma, implicit biases, and knowledge across Hong Kong, the United Kingdom and the United States, after controlling for relevant covariates using analyses of covariance (ANCOVAs, with the *aov()* function). For all analyses, we used a *p* < 0.05 significant threshold to determine whether to accept or reject the null hypothesis.

## Results

### Explicit stigma

A median explicit stigma score of −12 was found across the three countries. A Wilcoxon signed-rank test (using the *wilcox.test()* function) demonstrated that this value was significantly lower than zero (the neutral point on the stigma scale; *V* = 648.5, *p* < 0.0001). Notably, this score should not be interpreted as evidence of positive attitudes as it suggests that participants, on average, still expressed some discomfort around engaging with autistic people on at least one of the questions (see also S. C. Jones, 2022).

### Implicit biases

A median IAT D-score of 0.75 was found across the three groups. A Wilcoxon signed-rank test (using the *wilcox.test()* function) demonstrated that this value was significantly higher than zero (*V* = 62,940, *p* < 0.0001), indicating that participants responded more quickly when autism labels were paired with unpleasant attributes compared to when these attributes were paired with non-autistic labels. As such, participants’ implicit attitudes towards autism were clearly negative (replicating Dickter et al., 2020).

### Determining which factors contributed to explicit stigma, implicit biases, and knowledge

First, we aimed to determine which factors contributed to explicit stigma. Despite attempts to transform the data, parametric assumptions could not be met. Thus, we conducted a non-parametric multiple regression (using the *gam()* function) with age, gender, course level, autism-related knowledge, vertical individualism, horizontal individualism, vertical collectivism, horizontal collectivism, and implicit biases as predictors. We included age, gender, and course level as covariates here because previous research has found a contribution of these variables to autism-related stigma (Gillespie-Lynch et al., 2015; Kim, 2020; Kim et al., 2023; Kuzminski et al., 2019). Our analysis revealed that autism knowledge (*t*(345) = −10.46, *p* < 0.0001) and horizontal collectivism (*t*(345) = −2.03, *p* = 0.0428) were negative predictors, and vertical individualism (*t*(345) = 6.35, *p* < 0.0001) and implicit biases (*t*(345) = 2.17, *p* = 0.0306) were positive predictors, of explicit stigma. Those with less accurate autism-related knowledge, lower horizontal collectivism, higher vertical individualism, and higher implicit biases displayed greater levels of explicit stigma. There were no other significant predictors of explicit stigma (*p* > 0.05).

Next, we aimed to determine which factors contributed to implicit biases. After transforming the data (using the natural logarithm function), we conducted a multiple linear regression (using the *lm()* function) with age, gender, course level, autism-related knowledge, vertical individualism, horizontal individualism, vertical collectivism, horizontal collectivism, and explicit stigma as predictors. This analysis revealed that explicit stigma (*t*(345) = 2.17, *p* = 0.0306) and age (*t*(345) = 2.27, *p* = 0.0237) were positive predictors of implicit biases: those older in age and those who reported higher levels of explicit stigma, exhibited greater implicit biases. There were no other predictors (all *p* > 0.05).

Finally, to establish which factors contributed to autism knowledge, we conducted a multiple linear regression (using the *lm()* function, as parametric assumptions were met) with age, gender, course level, vertical individualism, horizontal individualism, vertical collectivism, and horizontal collectivism as predictors. This revealed that vertical individualism (*t*(347) = −3.05, *p* = .0025) and vertical collectivism (*t*(347) = −2.39, *p* = 0.0175) were negative predictors, and horizontal individualism (*t*(347) = 3.45, *p* = 0.0006) and horizontal collectivism (*t*(347) = 4.38, *p* < 0.0001) were positive predictors, of autism-related knowledge: individuals with greater acceptance of inequality (i.e. higher verticality and reduced horizontality) displayed less accurate autism-related knowledge. Our analyses also identified a significant effect of gender (*F*(5, 347) = 2.65, *p* = 0.0228). Post hoc *t* tests revealed that non-binary individuals (mean (standard error of the mean; SEM)) = 128.67 (2.12)) had significantly higher levels of autism-related knowledge than those who preferred not to disclose their gender (mean (SEM) = 107.83 (5.68); *t*(10) = 3.20, *p*_bonf_ = 0.0225). There were no other significant predictors of knowledge (all *p* > 0.05).

### Comparing attitudes towards, and knowledge about, autism across countries after controlling for relevant factors

Next, we aimed to compare levels of explicit stigma, implicit biases, and autism-related knowledge across countries, after controlling for variables that were significant predictors of these factors. We controlled for these significant predictors for three main reasons: (1) to ensure that any differences in explicit stigma, implicit biases, and/or knowledge between countries were not confounded by variation in the predictors across countries (e.g. different cultural orientations, different levels of knowledge); (2) to reduce omitted variable bias – in which the effects of omitted variables are mistakenly attributed to the predictors included in the model (Wilms et al., 2021; Wooldridge, 2012); and (3) to improve the precision of the estimated coefficients by accounting for additional sources of variation, leading to more reliable statistical inferences (Deaton & Cartwright, 2018; Hernández et al., 2004).

#### Explicit stigma

**Figure 1.**
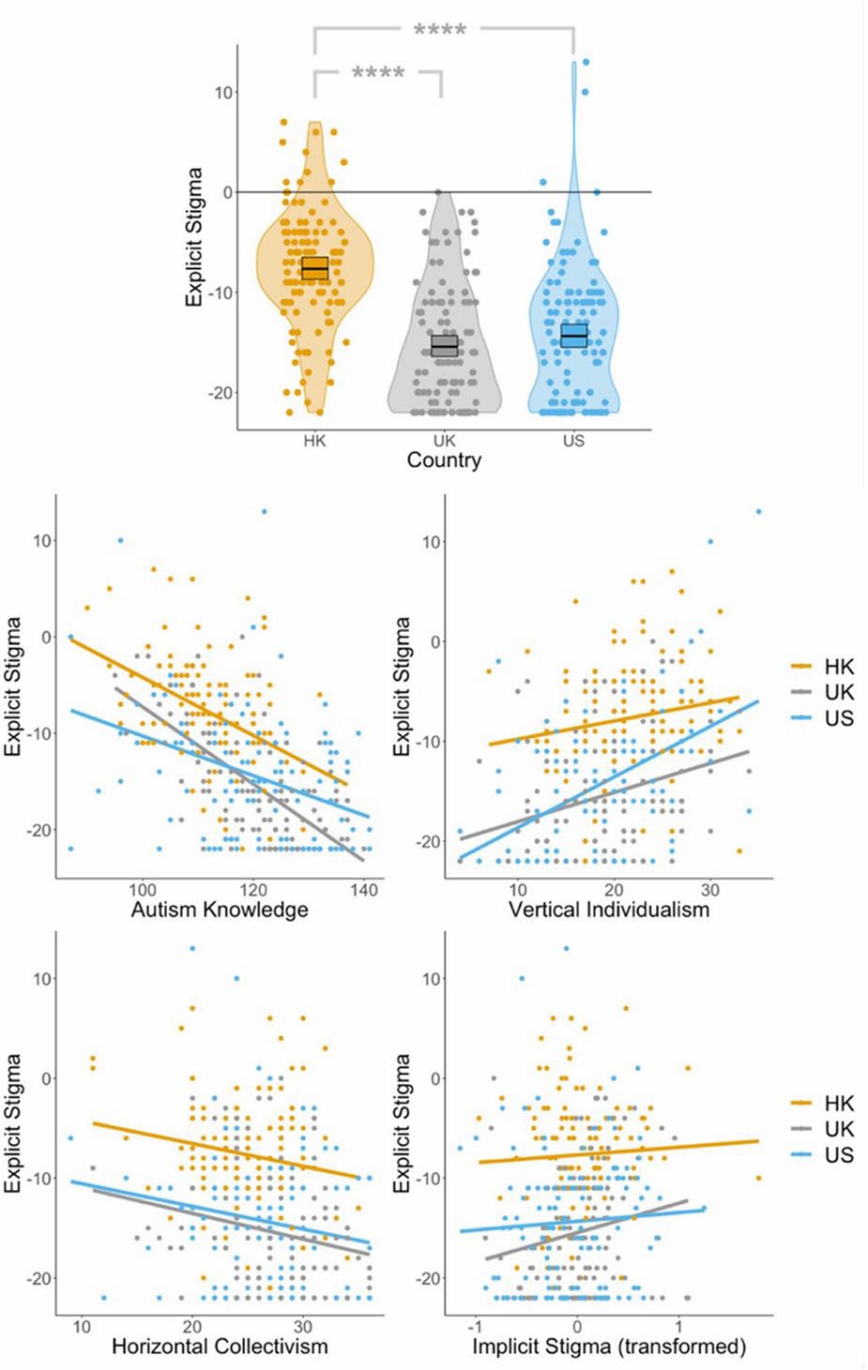
Plots displaying (1) levels of explicit stigma across Hong Kong, the United Kingdom, and the United States (top); (2) the relationships between explicit stigma and autism knowledge (middle-left), vertical individualism (middle-right), horizontal collectivism (bottom-left), and implicit biases (bottom-right) respectively, across Hong Kong, the United Kingdom, and the United States.

To determine whether there were differences in levels of explicit stigma between countries after controlling for knowledge, horizontal collectivism, vertical individualism and implicit biases (which were found to be significant predictors), we completed an ANCOVA (using the *aov()* function) on ranked data. This revealed a main effect of country (*F*(2, 354) = 22.12, *p* < 0.0001), which persisted even after accounting for the covariates. Post hoc Bonferroni-corrected Wilcoxon rank sum tests (using the *wilcox.test()* function) demonstrated that there were significantly higher levels of explicit stigma in Hong Kong than in the United Kingdom (*W* = 11,656, *p*_bonf_ < 0.0001) and the United States (*W* = 11,519, *p*_bonf_ < 0.0001), but no differences between these latter countries (*W* = 6676, *p*_bonf_ = 0.703; see Figure 1, top). In our ANCOVA, autism-related knowledge (*F*(1, 354) = −98.24, *p* < 0.0001) and horizontal collectivism (*F*(1, 354) = −4.28, *p* = 0.0392) were negative predictors, and vertical individualism (*F*(1, 354) = 33.00, *p* < 0.0001) and implicit biases (*F*(1, 354) = 5.45, *p* = 0.0201) were positive predictors, of explicit stigma (after accounting for country). In sum, those with lower levels of autism knowledge, lower horizontal collectivism, higher vertical individualism, higher implicit biases, and those in Hong Kong (relative to the United Kingdom and the United States) displayed greater levels of explicit stigma (see Figure 1).

After inspecting Figure 1 (bottom-right), we noted that the strength of the relationship between explicit stigma and implicit biases appeared to differ slightly across countries. As such, we added an implicit biases × country interaction to our previous ANCOVA. This interaction was not significant (*p* = 0.419). Thus, there were no significant differences in the strength of the relationship between explicit stigma and implicit biases across countries.

#### Implicit biases

**Figure 2.**
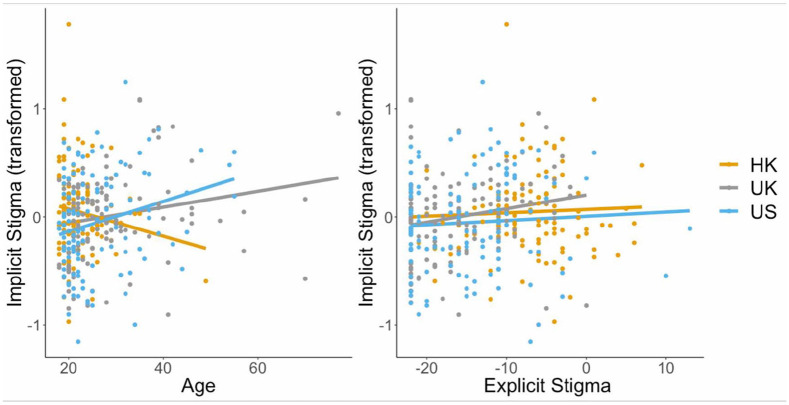
Scatterplots displaying the relationship between implicit biases and age (left), and implicit biases and explicit stigma (right) across Hong Kong, the United Kingdom, and the United States.

Next, to determine whether there were differences in levels of implicit biases between countries after controlling for age and explicit stigma (which were found to be significant predictors), we completed an ANCOVA (using the *aov()* function). In this analysis, there was no main effect of country (*F*(2, 354) = 0.86, *p* = 0.422). Only explicit stigma (*F*(1, 354) = 4.57, *p* = 0.0332) and age (*F*(1, 354) = 9.42, *p* = 0.0023) were significant predictors of implicit biases (see Figure 2).

By inspecting the scatterplot depicting the relationship between implicit biases and age (Figure 2, left), it became apparent that this relationship may be moderated by country. Therefore, we added an age × country interaction to our previous ANCOVA. This interaction was significant (*F*(2, 352) = 3.57, *p* = 0.0293): while implicit biases increased with growing age in the United Kingdom (*R* = 0.20, *p* = 0.0305) and the United States (*R* = 0.26, *p* = 0.0047), there was no relationship between implicit biases and age in Hong Kong (*R* = −0.14, *p* = 0.1178).

#### Knowledge

**Figure 3.**
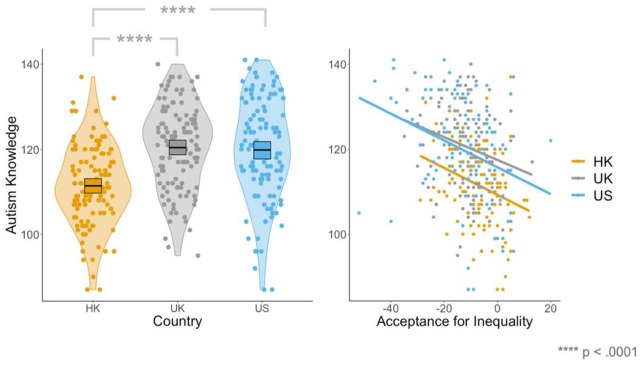
Plots displaying (1) levels of autism-related knowledge across Hong Kong, the United Kingdom and the United States (left), and (2) relationships between autism knowledge and acceptance of inequality across Hong Kong, the United Kingdom, and the United States.

Finally, to determine whether there were differences in levels of autism-related knowledge between countries after controlling for gender, vertical individualism, vertical collectivism, horizontal individualism, and horizontal collectivism (which were found to be significant predictors), we completed an ANCOVA (using the *aov() function*). This analysis revealed a significant main effect of country (*F*(2, 349) = 16.64, *p* < 0.0001), that persisted even after controlling for the covariates. Post hoc Bonferroni-corrected independent samples *t* tests (using the *t.test()* function) demonstrated that university students in Hong Kong displayed significantly lower levels of knowledge than in the United Kingdom (*t*(237) = −7.16, *p*_bonf_ < 0.0001) and the United States (*t*(239) = −6.09, *p*_bonf_ < 0.0001; see Figure 3, left). There were no significant differences in levels of knowledge between the United Kingdom and the United States (*t*(240) = 0.40, *p*_bonf_ = 1.000). In our ANCOVA, we found that vertical collectivism (*F*(1, 349) = −11.22, *p* = 0.0009) was a negative predictor, and horizontal collectivism (*F*(1, 349) = 18.09, *p* < 0.0001) and horizontal individualism (*F*(1, 349) = 5.87, *p* = 0.0159) were positive predictors, of autism-related knowledge. In sum, those with greater acceptance for inequality (i.e. higher verticality and lower horizontality), and those from Hong Kong (relative to the United Kingdom and the United States) displayed less accurate knowledge about autism.

### Exploratory mediation analyses

**Figure 4.**
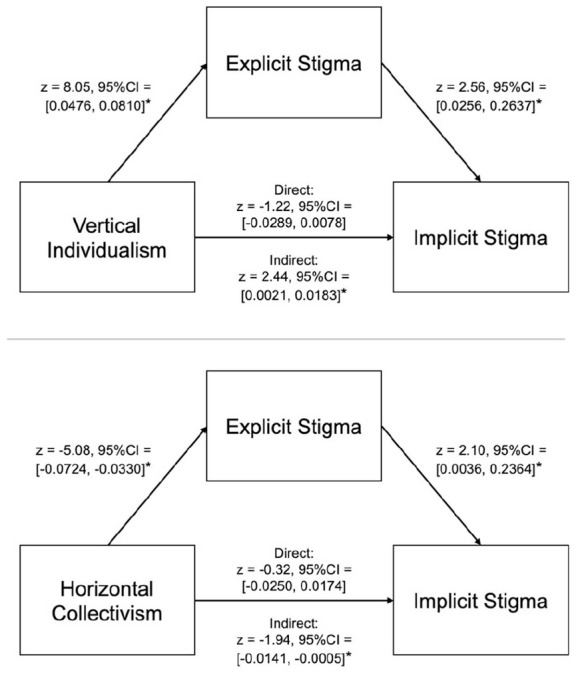
Mediation models showing the contribution of individualism (top) and acceptance of inequality (bottom) to implicit biases via explicit stigma. *Indicates that the confidence intervals do not cross zero, and therefore that the path is significant.

Finally, since we had identified that knowledge, vertical individualism and horizontal collectivism contributed to explicit stigma, which in turn contributed to implicit biases, we conducted three post hoc, exploratory mediation analyses to test for indirect effects. This approach was important to determine whether explicit stigma acts as a mediating pathway through which culture and knowledge contribute to implicit biases, offering deeper insight into the mechanistic links between these variables. Here, we used SEM for our mediation analyses, rather than standard regression models (Baron & Kenny, 1986), as SEM is regarded ‘a more appropriate inference framework for mediation analyses’ (Gunzler et al., 2013, p. 391). We employed bias-corrected bootstrapping (with 5000 replications) to create confidence intervals (CIs) as there is a consensus that this is the most powerful method for testing mediated effects (Cheung, 2007; Fritz & MacKinnon, 2007; Hayes & Scharkow, 2013; MacKinnon et al., 2004; Valente et al., 2016). If these CIs do not cross zero, there is evidence for the experimental hypothesis; if these CIs cross zero, there is evidence for the null hypothesis (Lane et al., 2013). In our three models, the predictors were knowledge, vertical individualism, and horizontal collectivism, respectively, the mediator was explicit stigma, and the outcome variable was implicit biases. These analyses revealed significant indirect effects of vertical individualism (*z* = 2.44, 95% CI = 0.0021, 0.0183) and horizontal collectivism (*z* = −1.94, 95% CI = −0.0141, −0.0005) on implicit biases, via explicit stigma (vertical individualism: *z* = 8.05, 95% CI = 0.0476, 0.0810; horizontal collectivism: *z* = −5.08, 95% CI = −0.0724, −0.0330; see Figure 4): those higher in vertical individualism, and those lower in horizontal collectivism, exhibited elevated levels of explicit stigma, which in turn led to higher levels of implicit biases. Conversely, there was not a significant indirect effect of knowledge on implicit biases (*z* = −1.46, 95% CI = −0.0116, 0.0015). One interpretation of these results is that our culture shapes our explicit attitudes and behaviours by providing a set of ‘social rules’ to follow. For example, horizontal collectivistic cultures encourage people to report equitable attitudes towards others. Over time, repeatedly engaging with these positive explicit attitudes could alter underlying implicit attitudes, resulting in the (implicit) belief that all members are equal (see Discussion for a full interpretation).

## Discussion

In this study, we first aimed to determine the contribution of knowledge and cultural orientation (among other factors) to *both* explicit stigma and implicit biases, and second, to compare levels of knowledge, explicit stigma, and implicit biases across Hong Kong, the United Kingdom, and the United States, after controlling for relevant covariates. Regarding our former aim, we found that higher explicit stigma was predicted by less accurate knowledge, greater vertical individualism, lower horizontal collectivism, and higher implicit biases. As such, our results add to a growing body of literature suggesting reduced knowledge may (at least in part) underlie heightened autism-related stigma (e.g. Gillespie-Lynch et al., 2015, 2019, 2022; Jones, DeBrabander, & Sasson, 2021a; Jones, Morrison, et al., 2021b; Kim et al., 2022; Kitchin & Karlin, 2022; Kuzminski et al., 2019; Ling et al., 2010; Obeid et al., 2015; Saade et al., 2021). Mechanistically, this inaccurate knowledge may result in individuals relying on reductionist steoretypes, dichotomizing into ‘them versus us’, and behaving in a discriminatory manner (Turnock et al., 2022). Beyond this, here we replicate previous findings suggesting that explicit stigma is predicted by greater vertical individualism (i.e. seeing the self as autonomous within a justly hierarchical system) and lower horizontal collectivism (i.e. not seeing the self as part of a collective whose members should be equal; Gillespie-Lynch et al., 2019; Kim et al., 2022), across all three countries studied.

Moreover, here, we found an association between explicit stigma and implicit biases, suggesting a degree of concordance between self-reported explicit attitudes, and underlying implicit attitudes, about autism. Visually, this association appeared to be strongest in the United Kingdom, but we observed no interaction with country. This finding contributes to the mixed literature on this topic: while some previous studies have found relationships between explicit and implicit measures of autism-related stigma (e.g. Kim et al., 2023, combined sample), others have not (Dickter et al., 2020; Jones, DeBrabander, & Sasson, 2021a). However, research outside of the autism literature suggests that IAT-based and self-report measures of attitudes towards groups are often associated (e.g. Hofmann et al., 2005).

Relatedly, we identified other direct and indirect relationships with implicit biases. With respect to the former, we found a (direct) positive association between implicit biases and age in the United Kingdom and the United States (but not Hong Kong): older adults typically displayed higher levels of implicit biases than younger adults. This finding is consistent with the results obtained by Kim et al. (2023) among South Koreans and it aligns with previous research documenting more pronounced biases regarding age (Chopik & Giasson, 2017), race (Gonsalkorale et al., 2009; Stewart et al., 2009), and disability (Goreczny et al., 2011), among older adults compared to younger ones.

Beyond this, our findings provide a novel contribution to the literature by elucidating factors that exert *indirect* effects on implicit biases via explicit stigma: vertical individualism and horizontal collectivism. An important theoretical question concerns how this may occur. From a mechanistic standpoint, culture may influence our explicit attitudes and behaviours by providing a set of ‘social rules’ to follow. For example, being immersed in a horizontal collectivistic culture – wherein the self is part of a collective whose members are seen as equal – encourages individuals to *report* equitable (explicit) attitudes towards others in order to preserve their social standing (or to avoid backlash from other members). Downstream, repeatedly engaging with these positive attitudes may extinguish and/or change the more durable, underlying beliefs (i.e. implicit biases) that have been reinforced over time. Thus, our cultural orientation may provide a set of ‘social rules’ that shape our explicit attitudes, which in turn influence our implicit attitudes. Although this is a logical possibility, given that SEM cannot definitively determine causality and directionality (Bollen & Pearl, 2013), further research employing longitudinal methods and causal manipulation is necessary (e.g. by testing how cultural orientation, explicit and implicit attitudes change after people move residence to a different culture) to confirm the nature of these relationships.

In this study, we also found that reduced autism-related knowledge was predicted by greater acceptance of inequality (i.e. higher verticality and lower horizontality), thus replicating previous results (Gillespie-Lynch et al., 2019). There are many potential explanations for this finding. On one hand, it could be that individuals who are more accepting of inequality are less inclined to seek out information or engage with marginalized groups with less power, resulting in poorer autism-related knowledge. On the other hand, the reverse direction of causality is possible: it may be that reduced or inaccurate knowledge about marginalized conditions and groups (e.g. autism and autistic people) predisposes individuals to be more accepting of inequality, as individuals rely on stereotypes and misconceptions which may paint these groups in a negative light. Alternatively, there could be a cyclical relationship, wherein reduced knowledge about marginalized groups (based on stereotypes and misconceptions) predisposes individuals to be more accepting of inequality, which in turn reduces their motivation to seek out information or engage with marginalized groups (thus maintaining incomplete or inaccurate knowledge), and so on. On a broader scale, it could be that fewer autism awareness campaigns – and/or autism awareness campaigns that spread less accurate or negative information about autism – are conducted within cultures with vertical orientations where unequal power distributions are more generally accepted or unquestioned by the public, organizations, and government, leading to reduced or inaccurate autism-related knowledge. Further work is necessary to ascertain the mechanisms underpinning the link between greater acceptance of inequality and limited autism-related knowledge.

Regarding our second aim, we identified significant cross-cultural variation in levels of autism-related knowledge and explicit stigma. Specifically, we found that there were lower levels of knowledge about, and greater (self-reported) explicit stigma towards, autism in Hong Kong than in the United Kingdom and the United States. These findings complement and extend previous results documenting greater knowledge, and lower explicit stigma, in the United Kingdom or United States than in South Korea (Kim et al., 2022), Japan (Keating et al., 2024; Someki et al., 2018), Lebanon (Gillespie-Lynch et al., 2019; Obeid et al., 2015), and Malaysia (de Vries et al., 2020).

Notably, the cross-cultural variation discovered here persisted even after controlling for relevant covariates (e.g. cultural orientation, gender, knowledge). This suggests that there may be additional factors that underpin the reduced or inaccurate knowledge and heightened stigma in Hong Kong, such as reduced openness to experience, or reduced presence of the neurodiversity movement (among other factors). With respect to the former, since being less open to new ideas and experiences is associated with higher explicit stigma (Gillespie-Lynch et al., 2019), and given that lower openness has been found in Hong Kong than in the United Kingdom (Chan, 2021), the elevated stigma in Hong Kong may be underpinned by lower openness to experience (see The Big Five Personality Traits; Digman, 1990). With respect to the latter, in Hong Kong, the dominant narratives shaping autism research and services are based on the medical model of deficits (Kwok & Kwok, 2020). While the endorsement of the neurodiversity movement in English-speaking Western countries has been associated with a positive shift in attitudes towards autism (den Houting, 2019; Kim & Gillespie-Lynch, 2023), limited exposure to the neurodiversity movement may contribute to the heightened stigma in Hong Kong (Cheng et al., 2023; Kim & Gillespie-Lynch, 2023; Turnock et al., 2022). Further research is necessary to determine the ‘additional’ factors that underpin cross-cultural variation in autism-related stigma and knowledge. By identifying these ‘additional’ factors, researchers will be able to construct comprehensive models that explain large amounts of variance in autism-related knowledge and stigma, paving the way for holistic interventions targeting multiple, highly influential factors.

An important finding from this study is that, although *explicit* stigma was higher in Hong Kong than in the United Kingdom and United States, there were no differences in *implicit* biases across countries. There are a number of potential interpretations of this finding. On one hand, it could be that individuals in the United Kingdom and the United States *genuinely* hold more positive explicit attitudes about autism than those in Hong Kong – perhaps due to being immersed within a culture where social norms have shifted to encourage individuals to be more aware and accepting of autistic people – while underlying, implicit attitudes are more durable, and thus taking longer to change. From a more cynical perspective, it could be that respondents from the United Kingdom and the United States simply r*eported* more positive (explicit) attitudes as it is socially desirable in these countries to do so (more so than in Hong Kong), when in reality both explicit and implicit attitudes in these countries are negative. Although this latter interpretation is a possibility, here we found a positive relationship between explicit and implicit stigma, suggesting that the self-reported explicit attitudes reflect underlying implicit attitudes to some degree. Such a relationship would not be present if individuals provided false representations of their explicit attitudes about autism. As such, our results point towards genuine differences in explicit stigma, but not implicit biases, across countries.

Relatedly, our findings build upon those from Kim and colleagues (2023), who identified higher levels of implicit autism-related biases in South Korea than in the United States. Synthesizing these results, if disparities in implicit biases exist between South Korea and the United States but not between Hong Kong, the United Kingdom, and the United States, this could suggest higher levels of implicit biases in South Korea relative to both Hong Kong and the United Kingdom (in addition to the United States). Future research is necessary to confirm whether this is true.

### Limitations

By examining autism-related knowledge and stigma across several countries, our sample is more culturally diverse than those traditionally employed in autism research. However, it is important to note that the samples from each of our countries may not be representative of their broader population with respect to gender, ethnicity, spoken language, and cultural orientation. First, since our samples were predominantly made up of cisgender females (Hong Kong: 63.0%; the United Kingdom: 65.8%; the United States: 54.9%), who tend to report lower levels of explicit stigma than males (Gillespie-Lynch et al., 2019; though see Kim et al., 2022), it is possible that levels of stigma in the broader population are underestimated here. Second, given that our samples were primarily White in the United Kingdom (70.0%) and the United States (64.8%), and Chinese in Hong Kong (81.5%), our results may not be representative of other ethnic groups. Third, while 69.3% of Hong Kong residents rate their English skills as sufficient to average for daily use (Hong Kong Census and Statistics Department, 2024), our whole sample were fluent in English and thus may not fully reflect the broader population. Finally, since our study focused on university students, who are thought to be more individualistic than those in the general population (Gillespie-Lynch et al., 2019), it is likely that some of the cross-cultural effects that we attempted to investigate (e.g., individualism-collectivism) were dampened here. Thus, future work should compare levels of autism-related stigma and knowledge across countries using representative general population samples. Such work should attempt to dismantle barriers to inclusion, ensuring that the samples represent the full range of diversity in each of the countries studied, for example, with respect to age, gender, ethnicity, level of education, and socioeconomic status.

It is also important to acknowledge the differences in recruitment procedures across countries. As mentioned previously, participants from Hong Kong were recruited via University of Hong Kong mailing lists, while those from the United Kingdom and the United States were recruited through Prolific. Consequently, our Hong Kong sample comprises students from a single institution, and thus may reflect specific institutional attitudes and norms, whereas the UK and US samples include students from a range of universities, resulting in more diverse institutional influences. Further research is necessary to examine whether university-specific practices influence knowledge about or stigma towards autism, and to investigate these factors across a broader range of universities in Hong Kong.

Finally, there is also ongoing debate surrounding the use of IAT tasks. While a full review of this literature is beyond the scope of this article, one concern is that IATs are vulnerable to contextual influences (Han et al., 2010) – though it is possible that implicit biases are genuinely shaped by context (Guinote et al., 2010; Jost, 2019). Another concern is that executive functions appear to impact IAT performance (Calanchini et al., 2014; Ito et al., 2015; Klauer et al., 2010), suggesting that IAT scores may not be a pure and uncensored reflection of implicit biases. A broader theoretical critique is that implicit biases, as measured by the IAT, may reflect exposure to societal stereotypes more than personal prejudice (Karpinski & Hilton, 2001; G. Mitchell & Tetlock, 2017). Nevertheless, previous studies indicate that IAT scores can predict behaviour (Jost, 2019; Jost et al., 2009), and in this study, implicit and explicit biases towards autism were related. If IAT scores are more indicative of exposure to stereotypes than personal prejudice, this would shift how we interpret individual results; however, the IAT would, nonetheless, still be a valuable tool for understanding autism stigma within societies.

## Conclusion

Here, we aimed to (1) assess the contribution of knowledge and cultural orientation to both explicit stigma and implicit biases towards autism, and (2) compare levels of knowledge, explicit stigma, and implicit biases across Hong Kong, the United Kingdom, and the United States. Regarding our former aim, we found that explicit stigma was predicted by less accurate knowledge, lower horizontal collectivism, greater vertical individualism, and higher implicit biases. Implicit biases were *directly* predicted by age and explicit stigma, and *indirectly* predicted by vertical individualism (positively) and horizontal collectivism (negatively) via explicit stigma. Regarding our latter aim, we identified cross-cultural variation in autism-related knowledge and explicit stigma, but not implicit biases, even after accounting for covariates: students in Hong Kong displayed lower levels of knowledge about, and higher explicit stigma towards autism, than those in the United Kingdom and the United States. These findings underscore the crucial need to combat misconceptions about, and stigma towards autism, particularly in Hong Kong.

## Supplemental Material

sj-docx-1-aut-10.1177_13623613241290565 – Supplemental material for Factors underlying differences in knowledge, explicit stigma and implicit biases towards autism across Hong Kong, the United Kingdom and the United States Supplemental material, sj-docx-1-aut-10.1177_13623613241290565 for Factors underlying differences in knowledge, explicit stigma and implicit biases towards autism across Hong Kong, the United Kingdom and the United States by Yulin Cheng, Patrick Dwyer and Connor Tom Keating in Autism

sj-docx-2-aut-10.1177_13623613241290565 – Supplemental material for Factors underlying differences in knowledge, explicit stigma and implicit biases towards autism across Hong Kong, the United Kingdom and the United States Supplemental material, sj-docx-2-aut-10.1177_13623613241290565 for Factors underlying differences in knowledge, explicit stigma and implicit biases towards autism across Hong Kong, the United Kingdom and the United States by Yulin Cheng, Patrick Dwyer and Connor Tom Keating in Autism

## Notes

1. Identity-first terminology is used throughout, in deference to arguments that autism is often central to identity/selfhood and that person-first terminology can reflect stigma (Geelhand et al., 2023; Gernsbacher, 2017; Keating et al., 2023). We would add that the language debate in autism research extends beyond the choice of identity-first or person-first terminology, and that the use of identity-first terminology alone is insufficient to reduce stigma and prejudice if other negative and deficit language choices are used (Dwyer, 2022; Keating et al., 2023).
