# Supplementary material for: Factors underlying differences in knowledge, explicit stigma and implicit biases towards autism across Hong Kong, the United Kingdom and the United States
Source: Autism. 2024 Nov 2;29(4):868–83. doi: 10.1177/13623613241290565 (PMC11967188; doi:10.1177/13623613241290565)
Supplement: sj-docx-1-aut-10.1177_13623613241290565 – Supplemental material for Factors underlying differences in knowledge, explicit stigma and implicit biases towards autism across Hong Kong, the United Kingdom and the United States [file sj-docx-1-aut-10.1177_13623613241290565.docx]

**Supplementary Materials A – Participants’ ethnicities**

***Table 1.*** Participants’ self-reported ethnicities.

| Country | Ethnicity | Frequency |
| --- | --- | --- |
| Hong Kong | Asian | 3 |
|  | Brown | 1 |
|  | Chinese | 97 |
|  | Chinese Hong Kong | 2 |
|  | Hong Konger | 9 |
|  | Indian | 2 |
|  | Mixed | 1 |
|  | Mixed – East Asian and Chinese | 1 |
|  | Nepali | 1 |
|  | White | 2 |
|  | **Total** | **119** |
|  |  |  |
| UK | Arab | 2 |
|  | Asian | 8 |
|  | Bangladeshi | 1 |
|  | Black | 7 |
|  | British | 1 |
|  | Chinese | 5 |
|  | Indian | 1 |
|  | Malay | 1 |
|  | Mixed | 1 |
|  | Mixed – Black and White | 2 |
|  | Mixed – Black African and White European | 1 |
|  | Mixed – Black Caribbean and and White | 1 |
|  | Mixed - White, Latine, and Ashkenazi Jewish | 1 |
|  | Pakistani | 1 |
|  | Pakistani British | 1 |
|  | South Asian | 1 |
|  | White | 81 |
|  | White British | 2 |
|  | White English | 1 |
|  | Other | 1 |
|  | **Total** | **120** |
|  |  |  |
|  | Asian | 3 |
| US | Black | 10 |
|  | Chinese | 5 |
|  | European | 1 |
|  | Hispanic/Latine | 7 |
|  | Lak | 1 |
|  | Mixed | 1 |
|  | Mixed – Asian and White | 2 |
|  | Mixed – Black and Puerto Rican | 1 |
|  | Mixed – Black and Hispanic | 1 |
|  | Mixed – Black and White | 3 |
|  | Mixed – Chinese and Asian | 1 |
|  | Mixed – Korean and American | 1 |
|  | Mixed – Vietnamese and White | 1 |
|  | Mixed – White and Hispanic/Latine | 3 |
|  | South Asian | 2 |
|  | White | 79 |
|  | **Total** | **122** |
