# Supplementary material for: Factors underlying differences in knowledge, explicit stigma and implicit biases towards autism across Hong Kong, the United Kingdom and the United States
Source: Autism. 2024 Nov 2;29(4):868–83. doi: 10.1177/13623613241290565 (PMC11967188; doi:10.1177/13623613241290565)
Supplement: sj-docx-2-aut-10.1177_13623613241290565 – Supplemental material for Factors underlying differences in knowledge, explicit stigma and implicit biases towards autism across Hong Kong, the United Kingdom and the United States [file sj-docx-2-aut-10.1177_13623613241290565.docx]

**Supplementary Materials B – The contribution of various factors to explicit stigma, implicit biases, and knowledge within each country.**

***Explicit stigma***

First, we aimed to determine which factors contributed to explicit stigma in each of our country groups. Despite attempts to transform the data, parametric assumptions could not be met. Therefore, we conducted non-parametric multiple regressions (using he *gam()* function) with age, gender, course level, autism-related knowledge, vertical individualism, horizontal individualism, vertical collectivism, horizontal collectivism, and implicit biases as predictors.

*Hong Kong*

Our analysis revealed that autism knowledge was a significant negative predictor of explicit stigma in Hong Kong [t(106) = -4.88, p < .0001]: those with less accurate autism-related knowledge exhibited higher explicit stigma in Hong Kong. There were no other significant predictors [all p > .05].

*UK*

Our analysis identified that autism knowledge was a significant negative predictor [t(107) = -8.31, p < .0001], and vertical individualism [t(107) = 2.52, p = .0132] and implicit biases [t(107) = 2.26, p = .0257] were significant positive predictors of explicit stigma in the UK. Those with less accurate autism-related knowledge, higher vertical individualism, and higher implicit biases displayed higher explicit stigma in the UK. There were no other significant predictors [all p > .05].

US

Our analysis found that autism knowledge was a significant negative predictor [t(108) = -3.50, p = .0007], and vertical individualism [t(108) = 5.02, p < .0001] was a significant positive predictor of explicit stigma in the US. Those in the US with less accurate autism-related knowledge and higher vertical individualism displayed higher levels of explicit stigma. There were no other significant predictors [all p > .05].

***Implicit biases***

Second, we aimed to determine which factors contributed to implicit biases in each of our country groups. After transforming the data (using the natural logarithm function), we conducted a multiple linear regression (using the *lm()* function) with age, gender, course level, autism-related knowledge, vertical individualism, horizontal individualism, vertical collectivism, horizontal collectivism, and explicit stigma as predictors.

*Hong Kong*

Our analysis revealed that age was a significant negative predictor of implicit biases in Hong Kong [t(106) = -2.08, p = .0399]. Those older in age in Hong Kong displayed lower levels of implicit biases. There were no other significant predictors [all p > .05].

*UK*

Our analysis identified that explicit stigma was a significant positive predictor of implicit biases in the UK [t(107) = 2.26, p = .0257]: individuals in the UK who reported higher levels of explicit stigma showed greater implicit biases (against autism). There were no other significant predictors [all p > .05].

*US*

Our analysis identified that age was a significant positive predictor of implicit biases in the US [t(108) = 2.33, p = .0218]. Older individuals in the US displayed higher levels of implicit biases. There were no other significant predictors [all p > .05].

***Knowledge***

Finally, we aimed to determine which factors contributed to autism-related knowledge in each of our country groups. To do so, we conducted a multiple linear regression (using the *lm()* function, as parametric assumptions were met) with age, gender, course level, vertical individualism, horizontal individualism, vertical collectivism, and horizontal collectivism as predictors.

*Hong Kong*

Our analysis revealed that horizontal individualism was a significant positive predictor of knowledge [t(108) = 3.23, p = .0016]. There were no other significant predictors [all p > .05].

*UK*

Our analysis identified no significant predictors of autism-related knowledge [all p > .05].

*USA*

Our analysis found that vertical collectivism [t(110) = -3.43, p = .0008] and age [t(110) = -2.00, p = .0482] were negative predictors, and horizontal collectivism [t(110) = 2.94, p = .0040] was a positive predictor of autism-related knowledge. US individuals younger in age, with lower vertical collectivism, and higher horizontal collectivism had more accurate autism-related knowledge. In addition, we discovered an effect of course level [t(110) = -2.10, p = .0382], in which postgraduate students [mean(SEM) = 123(3.23)] had more accurate autism-related knowledge than undergraduate students [mean(SEM) = 118(2.65)]. There were no other significant predictors [all p > .05].
